# Crystal structure and Hirshfeld surface analysis of 8-aza­niumylquinolinium tetra­chlorido­zincate(II)

**DOI:** 10.1107/S2056989023007466

**Published:** 2023-08-30

**Authors:** Gulnora A. Umirova, Khayit Kh. Turaev, Bekmurod Kh. Alimnazarov, Sherzod A. Kasimov, Abdulakhat T. Djalilov, Bakhtiyar T. Ibragimov, Jamshid M. Ashurov

**Affiliations:** a Termez State University, Barkamol avlod street 43, Termez city, Uzbekistan; b Tashkent Scientific Research Institute of Chemical Technology, Township Shura-bazar, District of Zangiata, Tashkent 111116, Uzbekistan; c Institute of Bioorganic Chemistry, Academy of Sciences of Uzbekistan, M. Ulugbek Str. 83, Tashkent 700125, Uzbekistan; Vienna University of Technology, Austria

**Keywords:** 8-Amino­quinoline, inter­molecular inter­actions, crystal structure, hydrogen bonding, π–π stacking, Hirshfeld surface

## Abstract

The reaction of 8-amino­quinoline, zinc chloride and hydro­chloric acid in ethanol yielded the title salt, (C_9_H_10_N_2_)[ZnCl_4_], which consists of a planar 8-aza­n­ium­yl­quinolinium dication and a tetra­hedral tetra­chloro­zincate dianion. The 8-amino­quinoline moiety is protonated at both the amino and the ring N atoms. In the crystal, the cations and anions are connected by inter­molecular N—H⋯Cl and C—H⋯Cl hydrogen bonds, forming sheets parallel to (001). Adjacent sheets are linked through π–π inter­actions involving the pyridine and arene rings of the 8-aza­niumylquinolinium dication. Hirshfeld surface analysis indicates that the most important contributions to the crystal packing are from H⋯Cl (48.1%), H⋯H (19.9%), H⋯C/C⋯H (14.3%) (involving the cations) and H⋯Cl (82.6%) (involving the anions) interactions.

## Abstract 2

The organic–inorganic hybride salt (C_9_H_10_N_2_)[ZnCl_4_] consists of a planar 8-aza­niumylquinolinium dication and a tetra­hedral tetra­chlorido­zincate(II) dianion, held together by N—H⋯Cl and C—H⋯Cl hydrogen bonds, and π—π inter­actions.

## 1. Chemical context

Quinoline and its derivatives com­prise an important group of heterocyclic com­pounds that exhibit a wide range of pharmacological properties, such as anti­malarial (Shiraki *et al.*, 2011 ▸; Singh *et al.*, 2011 ▸; Murugan *et al.*, 2022 ▸), anti­bacterial (Upadhayaya *et al.*, 2009 ▸; Zeleke *et al.*, 2020 ▸), anti­microbial (Teja *et al.*, 2016 ▸), anti-inflammatory (Guirado *et al.*, 2012 ▸), anti­cancer (Abbas *et al.*, 2015 ▸), anti­diabetic (Kulkarni *et al.*, 2012 ▸) and anti­histaminic activities (Sridevi *et al.*, 2010 ▸). The quinoline moiety is found in many drugs and is useful in the rational design of novel bioactive mol­ecules in medicinal chemistry. The inter­est in 8-amino­quinoline, which contains functional groups commonly involved in hydrogen bonding, is related to its genotoxic activities, such as mutagenicity (Takahashi *et al.*, 1987 ▸), and to its unusually low proton-acceptor ability in solution. Quinolines are also strongly fluorescent and have been employed in the analytical study of heavy metals (Fritsch *et al.*, 2006 ▸). They have also been used to prepare highly conducting copolymers (Li *et al.*, 2005 ▸). As a ligand, 8-amino­quinoline usually binds in a bidentate fashion *via* the two N-atom positions (Setifi *et al.*, 2016 ▸; Mao *et al.*, 2018 ▸; Yang *et al.*, 2019 ▸), although examples of bridging–binding modes are also known (Schmidbaur *et al.*, 1991 ▸). In addition, 8-amino­quinoline can form π–π stacking inter­actions with (other) aromatic rings, thus controlling the inter­growth of inter­penetrating networks (Khelfa *et al.*, 2021 ▸; Rahmati *et al.*, 2018 ▸). Zinc, an essential com­ponent of life, is an abundant ion in living organisms (Andreini *et al.*, 2006 ▸; Cuajungco *et al.*, 2021 ▸). A bioinformatics study found that over 50% of zinc-bound proteins are enzymes, and in the vast majority of them, the metal plays a catalytic role (Andreini & Bertini, 2012 ▸). About 20% of them feature zinc as a structural com­ponent (Banci *et al.*, 2002 ▸; Andreini & Bertini, 2012 ▸). Zinc com­plexes exhibit a wide range of coordination numbers and coordination spheres, with tetra­hedral (Ashurov *et al.*, 2018 ▸; Petrus *et al.*, 2020 ▸) and octa­hedral (Ashurov *et al.*, 2011 ▸) environments being the most frequently observed. 
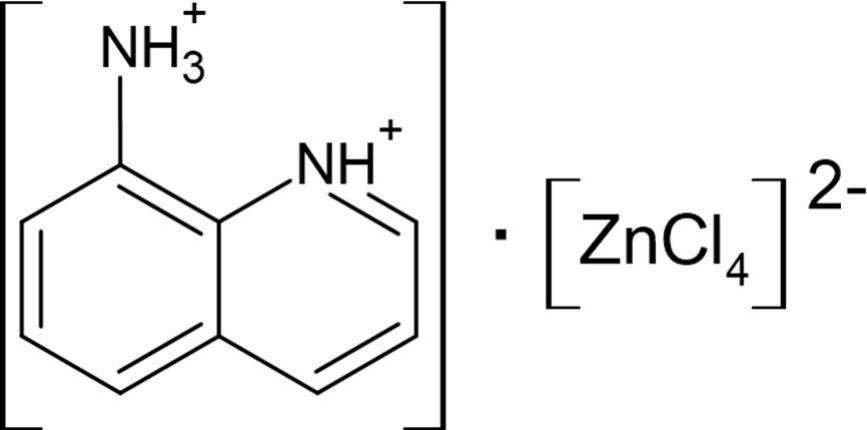


In the context given above, we report here the synthesis, crystal structure and Hirshfeld surface analysis of the organic–inorganic hybride salt (C_9_H_10_N_2_)[ZnCl_4_].

## 2. Structural commentary

**Figure 1:**
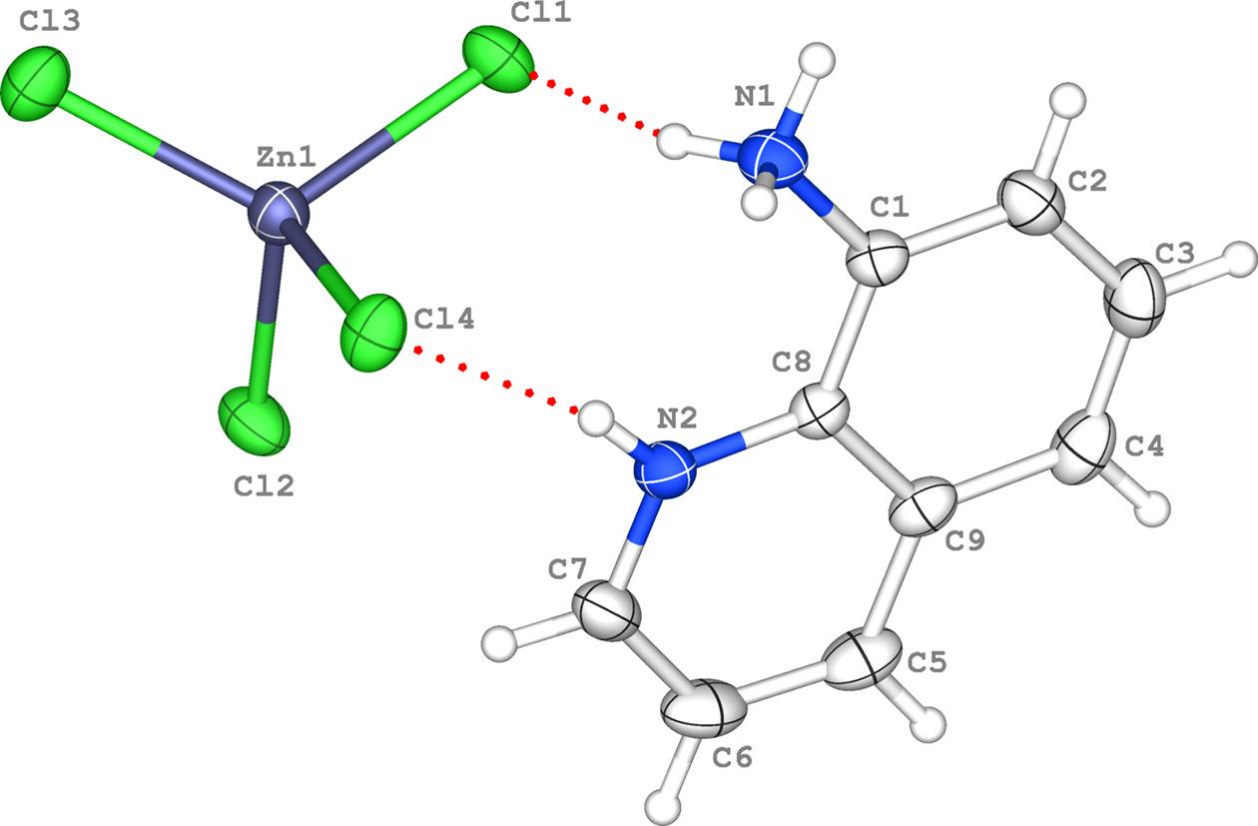
View of the asymmetric unit of the title salt, showing the atom-labelling scheme. Displacement ellipsoids are drawn at the 50% probability level. H atoms are shown as small spheres of arbitrary radius and hydrogen bonds are shown as dashed lines.

The title salt crystallizes with one (C_9_H_10_N_2_)^2+^ dication and one [ZnCl_4_]^2−^ dianion in the asymmetric unit (Fig. 1 ▸). The cation consists of an 8-amino­quinoline moiety that is proton­ated at both the amino and the ring N atoms. Protonation of the amino group results in a lengthening of the C—N(*sp* ^3^) bond from 1.377 (3) Å (*sp* ^2^ N) in 8-amino­quinoline (Van Meervelt *et al.*, 1997 ▸) to 1.464 (2) Å. This reflects the loss of the conjugation between the aromatic ring and the lone-pair electrons of the amino N atom when the latter is protonated. The quinoline ring system (atoms C1–C9/N2) is essentially planar; the r.m.s. deviation for the non-H atoms is 0.017 (2) Å, with a maximum deviation from the mean plane of 0.022 (2) Å for the C7 atom. The aza­niumyl N atom is almost coplanar with the quinoline plane, deviating from it by only 0.033 (2) Å. The coordination environment of the Zn atom in the [ZnCl_4_]^2−^ dianion is slightly distorted tetra­hedral (τ^4^ = 0.91; Yang *et al.*, 2007 ▸). The mean value of the Zn—Cl bond lengths of the [ZnCl_4_]^2−^ anion is 2.279 Å, which is in good agreement with the literature value [2.268 (4) Å; Harrison, 2005 ▸]. The Cl—Zn—Cl bond angles in the dianion indicate distortions from a regular tetra­hedron (109.5°), with a spread of values between 103.058 (19) and 117.08 (2)°. The most acute angle of 103.058 (19)° within the tetra­chlorido­zincate dianion is sub­tended by atoms Cl1 and Cl4. These atoms are associated with the relatively long Zn—Cl bond lengths, which, in turn, are correlated with the most relevant inter­molecular inter­actions in the structure; atom Cl4 is involved in the shortest and most linear N—H⋯Cl hydrogen bond (see Section 3) and thus represents the most distant ligand in the anion.

## 3. Supra­molecular features and Hirshfeld surface analysis

**Figure 2:**
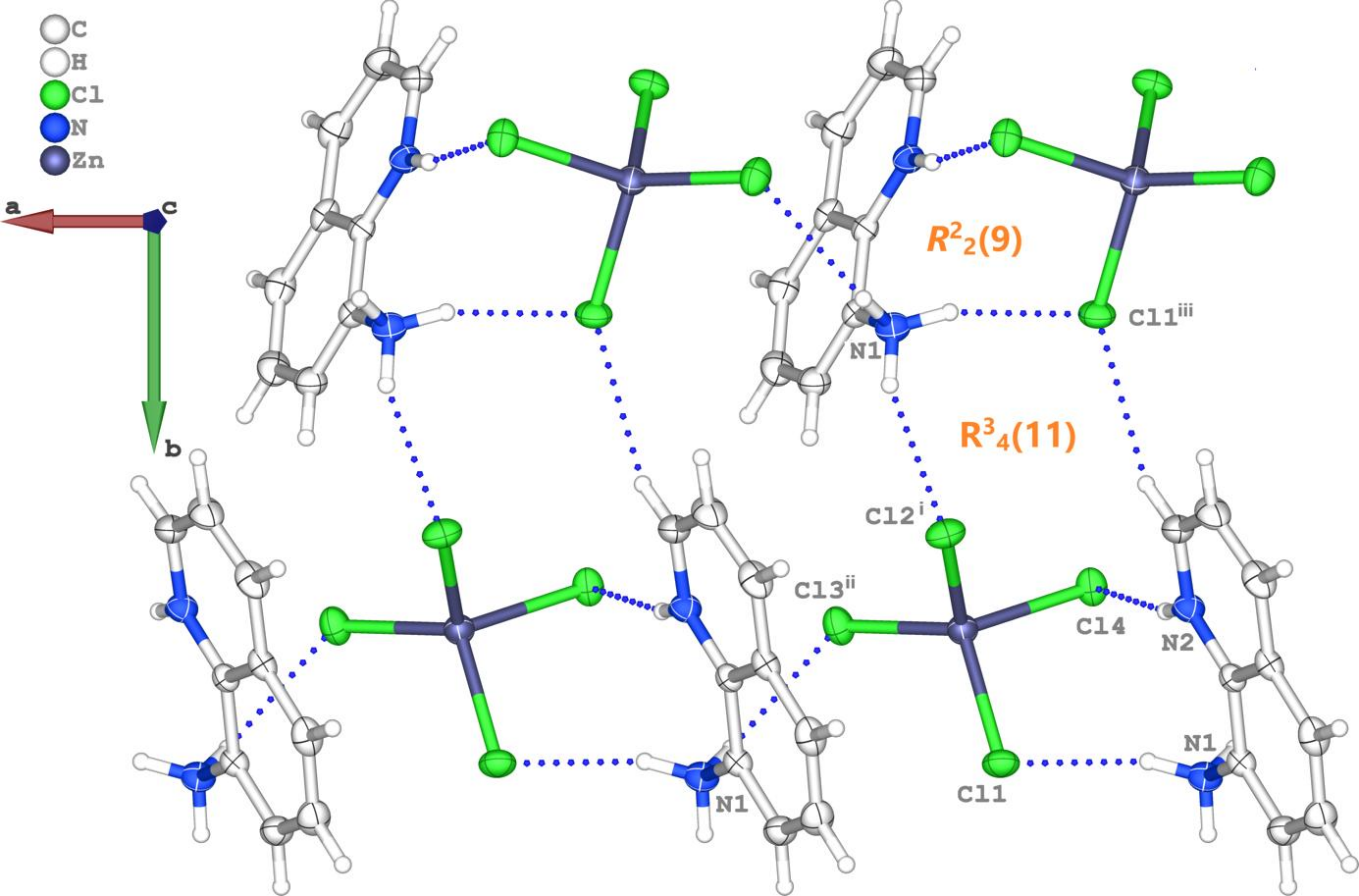
The formation of N1—H1*A*⋯Cl1, N1—H1*B*⋯Cl2^i^, N1—H1*C*⋯Cl3^ii^, N2—H2⋯Cl4 and C7—H7⋯Cl1^iii^ hydrogen bonds (dashed blue lines) in the crystal structure, leading to 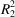 (9) and 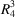 (11) graph-set motifs. The symmetry codes are as in Table 1 ▸.

**Figure 3:**
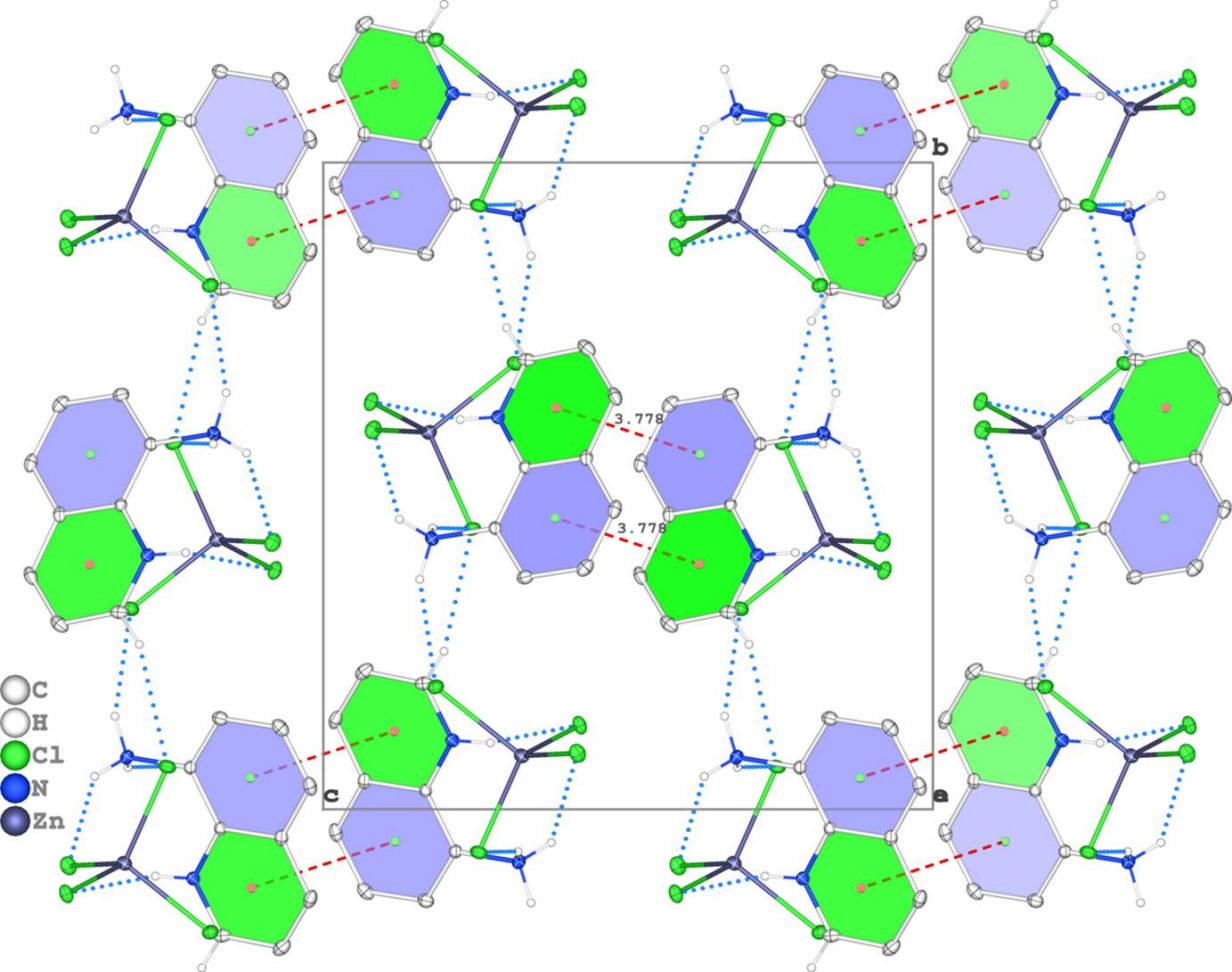
The crystal packing viewed along [100]. N—H⋯Cl and C—H⋯Cl hydrogen bonds are shown as blue dashed lines, while the π–π stacking inter­actions are shown as red dashed lines.

Each [ZnCl_4_]^2−^ dianion is connected to four neighbouring organic cations through N—H⋯Cl and C—H⋯Cl inter­actions involving all the Cl atoms (Table 1 ▸). Thus, the N1—H1*A*⋯Cl1, N2—H2⋯Cl4 and N1—H1*C*⋯Cl3^ii^ hydrogen bonds generate 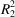 (9) ring motifs (Bernstein *et al.*, 1995 ▸) and link the dications and anions into chains parallel to [100] (Fig. 2 ▸). These chains are inter­connected by N1—H1*B*⋯Cl2^i^ and C7— H7⋯Cl1^iii^ hydrogen bonds, which generate 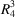 (11) ring motifs, forming sheets parallel to (001) (Fig. 2 ▸). In addition, the mol­ecules are linked by pairs of π–π inter­actions between the pyridine and arene rings of neighbouring dications. The mol­ecules stack along [001] to consolidate the triperiodic supra­molecular network (Fig. 3 ▸). The relevant centroid-to-centroid distance for π–π stacking inter­action between *Cg*1 (the centroid of pyridine ring C5–C7/N2/C8/C9) and *Cg*2 (the centroid of arene ring C1–C4/C9/C8) is *Cg*1⋯*Cg*2^i^ = 3.7784 (11) Å [symmetry code: (i) −*x* + 1, −*y* + 1, −*z* + 1], with a slippage of 1.613 Å.

**Figure 4:**
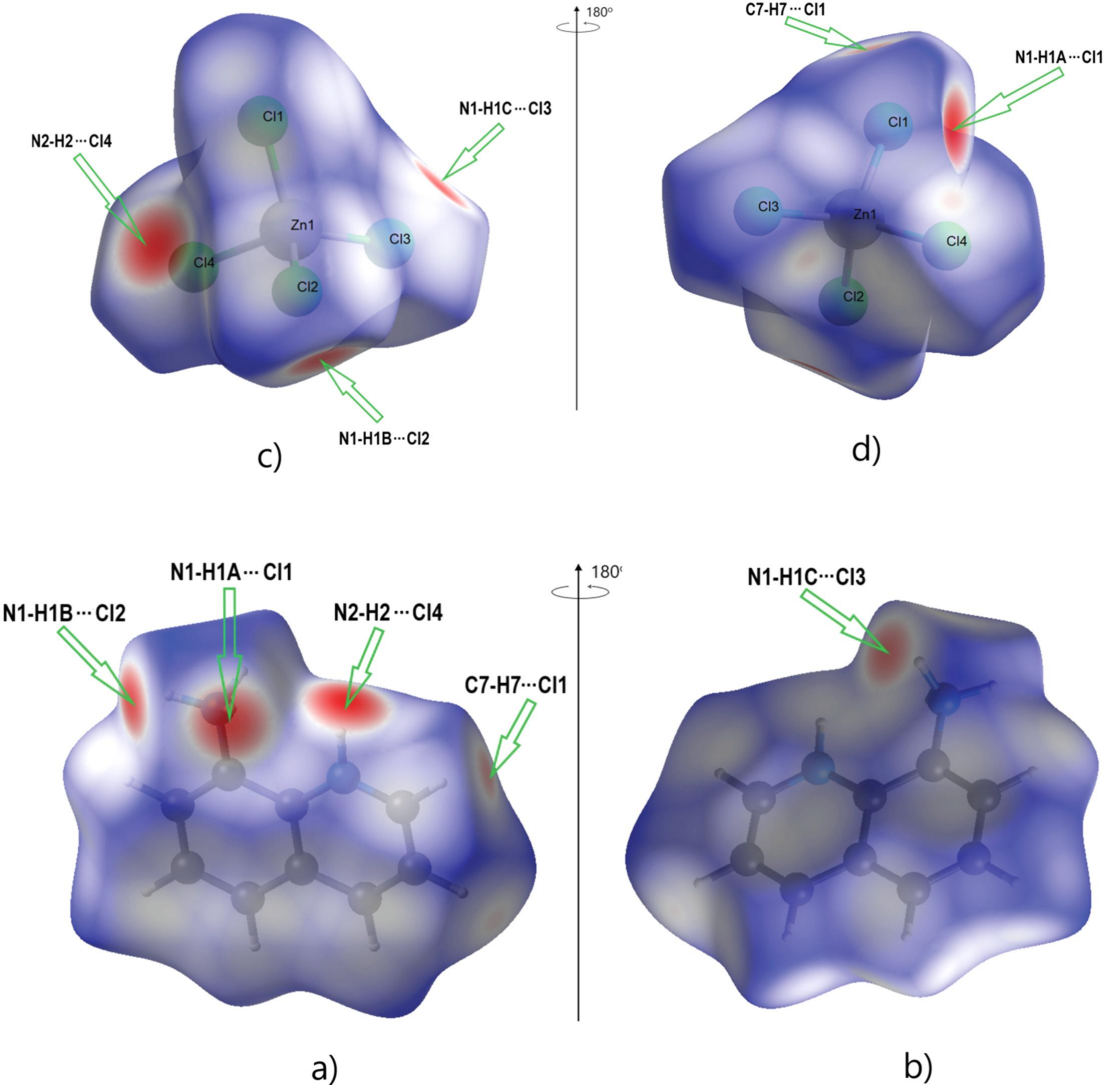
View of the three-dimensional Hirshfeld surface for the (C_9_H_10_N_2_)^2+^ dication and the [ZnCl_4_]^2−^ dianion plotted over *d*_norm_. Parts (*a*) and (*b*) show the front and back sides, respectively, of the (C_9_H_10_N_2_)^2+^ dication. Parts (*c*) and (*d*) show the front and back sides, respectively, of the [ZnCl_4_]^2−^ dianion.

**Figure 5:**
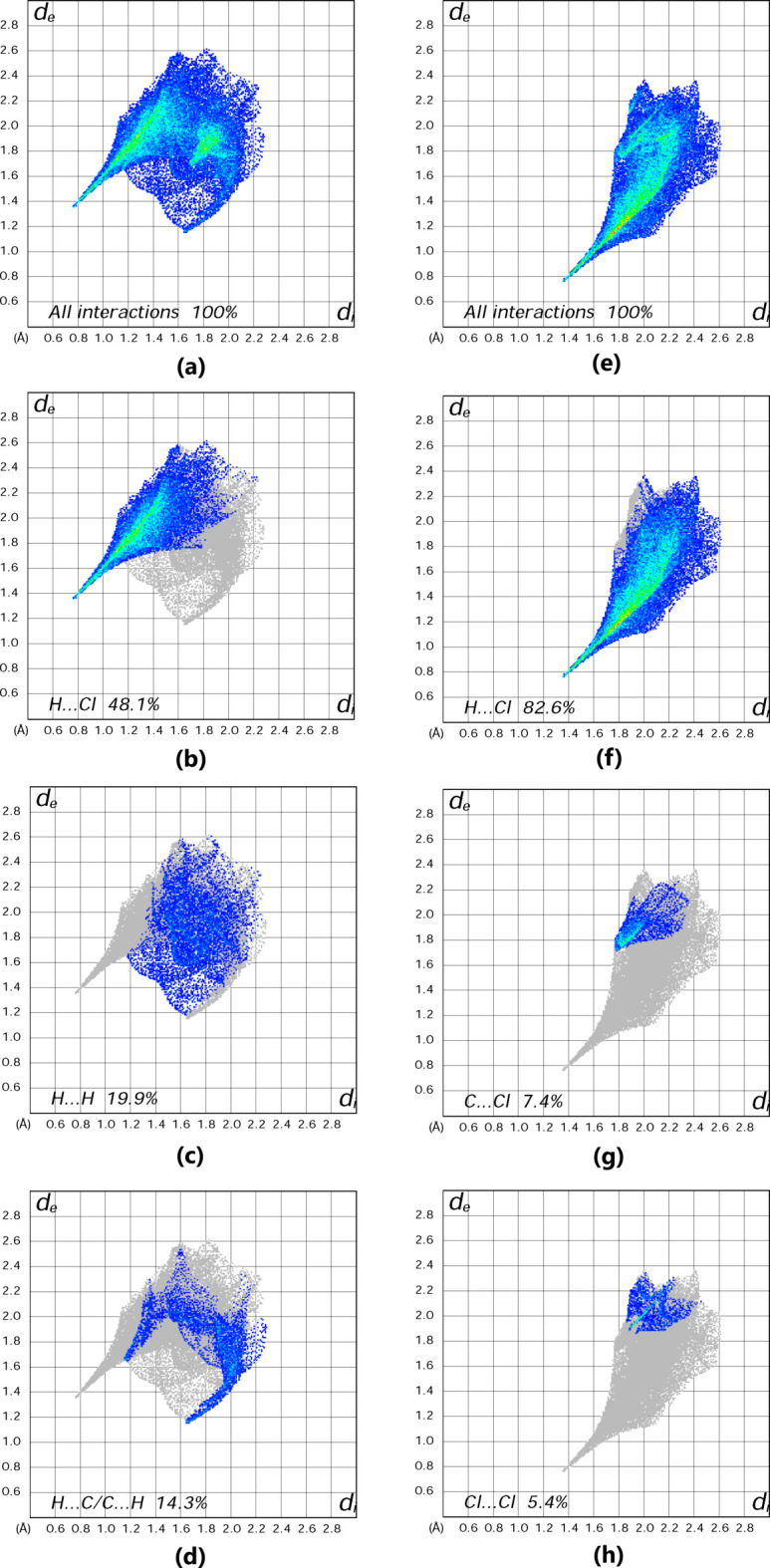
Two-dimensional Hirshfeld surface fingerprint plots for the (C_9_H_10_N_2_)^2+^ dication [panels (*a*), (*b*), (*c*) and (*d*)] and the [ZnCl_4_]^2−^ dianion [panels (*e*), (*f*), (*g*) and (*h*)]. The *d*_i_ and *d*_e_ values are the closest inter­nal and external distances (in Å) from a given point on the Hirshfeld surface.

**Figure 6:**
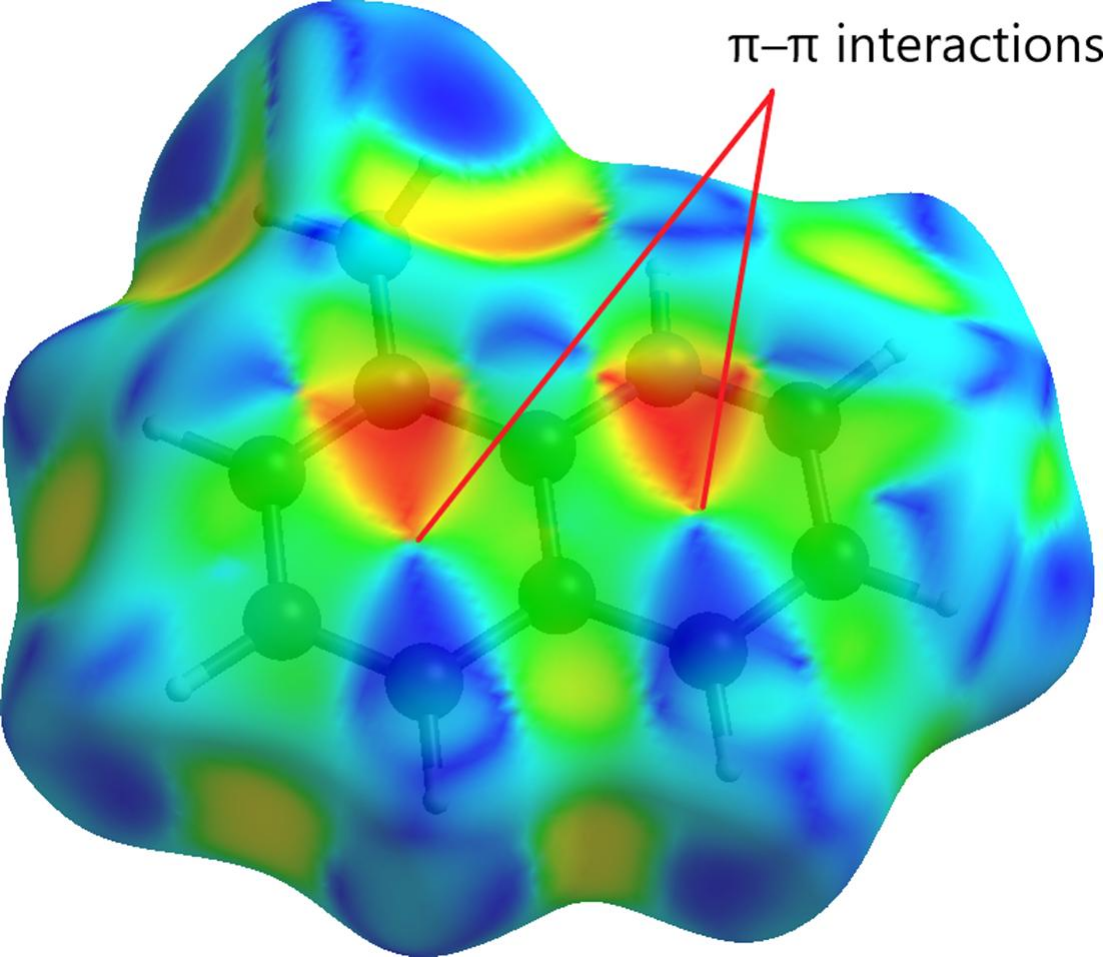
The Hirshfeld surface of the (C_9_H_10_N_2_)^2+^ dication plotted over shape-index.

The supra­molecular inter­actions were investigated qu­anti­tatively and visualized by Hirshfeld surface analysis per­form­ed with *CrystalExplorer21* (Spackman *et al.*, 2021 ▸). It should be noted that the Hirshfeld surfaces and fingerprint plots were calculated separately for the 8-aza­niumyl­quino­lin­ium dication and the [ZnCl_4_]^2−^ dianion. The respective acceptor and donor atoms showing strong N—H⋯Cl inter­molecular hydrogen bonds (for N1—H1*A*⋯Cl1, N1—H1*B*⋯Cl2^i^, N1—H1*C*⋯Cl3^ii^ and N2—H2⋯Cl4) are indicated as bright-red spots on the Hirshfeld surface (Fig. 4 ▸). Classical N—H⋯Cl hydrogen bonds correspond to H⋯Cl contacts [with contributions of 82.6 and 48.1% to the Hirshfeld surface for the [ZnCl_4_]^2−^ dianion and 8-aza­niumylquinolinium dication, respectively; Figs. 5 ▸(*f*) and 5(*b*)]. These inter­actions can be seen as spikes with a sharp tip. H⋯H, H⋯C/C⋯H and C⋯C inter­actions in the dication, and C⋯Cl and Cl⋯Cl inter­actions in the dianion follow with contributions of 19.9, 14.3, 6.7, 7.4 and 5.4%, respectively (Fig. 5 ▸). Other, minor, con­tributions are from C⋯Cl (6.4%), H⋯N/N⋯H (2.6%), H⋯Zn (0.7%), N⋯Cl (0.6%) and N⋯C/C⋯N (0.1%) contacts in the dication, and from Zn⋯Cl/Cl⋯Zn (1.7%), Zn⋯H (1.1%), N⋯Cl (1.0%) and Zn⋯C (0.8%) contacts in the dianion. The shape-index of the 8-aza­niumylquinolinium di­cation is a tool to visualize π–π stacking by the presence of adjacent red and blue triangles. Fig. 6 ▸ gives clear evidence that these inter­actions exist, as discussed above.

## 4. Database survey

A search of the Cambridge Structural Database (CSD, Version 2022.3.0; Groom *et al.*, 2016 ▸) revealed 114 com­pounds involving the 8-amino­quinoline moiety. Among them, 65 are metal com­plexes and 20 are organic salts and cocrystals. In all of these metal com­plexes, 8-amino­quinoline coordinates in a bidentate fashion, although there are examples of bridging–binding (CSD refcode VIZBIP; Schmidbaur *et al.*, 1991 ▸) and monodentate (MUDNEG; Xu *et al.*, 2015 ▸) modes. Only in the structure of 8-aza­niumylquinolinium dichloride (PENHAR; Yan *et al.*, 1998 ▸) are both the amino group and the ring N atom protonated.

## 5. Synthesis and crystallization

Commercially available starting materials were used without further purification. 8-Amino­quinoline (0.144 g, 1 mmol) was dissolved in 10 ml of an ethanol/HCl mixture (9:1 *v*/*v*) and added to a solution of ZnCl_2_ (0.136 g, 1 mmol) in 10 ml of the same ethanol/HCl mixed solvent. The mixture was heated under reflux and stirred for 30 min. A pale-yellow crystalline product was obtained at room temperature after 6 d by slow solvent evaporation [yield: 80%; elemental analysis calculated (%) for C_9_H_10_Cl_4_N_2_Zn: C 30.59, H 2.85, N 7.93; found: C 30.43, H 2.79, N 7.89].

## 6. Refinement

Crystal data, data collection and structure refinement details are summarized in Table 2 ▸. C-bound H atoms were placed in calculated positions and refined using the riding-model approximation, with *U*_iso_(H) = 1.2*U*_eq_(C) and C—H = 0.93 Å for aromatic H atoms. Both the amino and the ring N-bound H atoms were located in a difference Fourier map and refined with bond-length restraints of 0.89 (1) and 0.86 (1) Å, res­pectively.

## Supplementary Material

Crystal structure: contains datablock(s) I. DOI: 10.1107/S2056989023007466/wm5692sup1.cif

Structure factors: contains datablock(s) I. DOI: 10.1107/S2056989023007466/wm5692Isup2.hkl

CCDC reference: 2290822

Additional supporting information:  crystallographic information; 3D view; checkCIF report

## Figures and Tables

**Table 1:** Hydrogen-bond geometry (Å, °)

*D*—H⋯*A*	*D*—H	H⋯*A*	*D*⋯*A*	*D*—H⋯*A*
N1—H1*A*⋯Cl1	0.88 (1)	2.31 (1)	3.1309 (16)	154 (2)
N1—H1*B*⋯Cl2^i^	0.89 (1)	2.39 (1)	3.1747 (17)	148 (2)
N1—H1*C*⋯Cl3^ii^	0.88 (2)	2.48 (2)	3.2415 (16)	145 (2)
N2—H2⋯Cl4	0.86 (2)	2.25 (2)	3.0958 (16)	166 (2)
C7—H7⋯Cl1^iii^	0.93	2.71	3.584 (2)	157

Symmetry codes: (i) 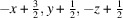 ; (ii) 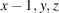 ; (iii) 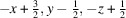 .

**Table 2:** Experimental details

Crystal data	
Chemical formula	(C_9_H_10_N_2_)[ZnCl_4_]
*M*_r_	353.36
Crystal system, space group	Monoclinic, *P*2_1_/*n*
Temperature (K)	566
*a*, *b*, *c* (Å)	7.52646 (6), 13.40703 (12), 12.65801 (11)
β (°)	92.8635 (8)
*V* (Å^3^)	1275.69 (2)
*Z*	4
Radiation type	Cu *K*α
μ (mm^−1^)	10.16
Crystal size (mm)	0.24 × 0.21 × 0.15

Data collection	
Diffractometer	Rigaku XtaLAB Synergy single source diffractometer with a HyPix3000 detector
Absorption correction	Multi-scan (*CrysAlis PRO*; Rigaku OD, 2020 ▸)
*T*_min_, *T*_max_	0.491, 1.000
No. of measured, independent and observed [*I* > 2σ(*I*)] reflections	11239, 2474, 2341
*R*_int_	0.037
(sin θ/λ)_max_ (Å^−1^)	0.615

Refinement	
*R*[*F* ^2^ > 2σ(*F* ^2^)], *wR*(*F* ^2^), *S*	0.023, 0.065, 1.06
No. of reflections	2474
No. of parameters	162
No. of restraints	4
H-atom treatment	H atoms treated by a mixture of independent and constrained refinement
Δρ_max_, Δρ_min_ (e Å^−3^)	0.32, −0.28

Computer programs: *CrysAlis PRO* (Rigaku OD, 2020 ▸), *SHELXT* (Sheldrick, 2015*a* ▸), *SHELXL* (Sheldrick, 2015*b* ▸), *OLEX2* (Dolomanov *et al.*, 2009 ▸) and *publCIF* (Westrip, 2010 ▸).

## supplementary crystallographic information

### Crystal data

**Table:**

(C_9_H_10_N_2_)[ZnCl_4_]	*F*(000) = 704
*M**_r_* = 353.36	*D*_x_ = 1.840 Mg m^−^^3^
Monoclinic, *P*2_1_/*n*	Cu *K*α radiation, λ = 1.54184 Å
*a* = 7.52646 (6) Å	Cell parameters from 8253 reflections
*b* = 13.40703 (12) Å	θ = 3.3–71.4°
*c* = 12.65801 (11) Å	µ = 10.16 mm^−^^1^
β = 92.8635 (8)°	*T* = 566 K
*V* = 1275.69 (2) Å^3^	Block, pale yellow
*Z* = 4	0.24 × 0.21 × 0.15 mm

### Data collection

**Table:**

Rigaku XtaLAB Synergy single source diffractometer with a HyPix3000 detector	2474 independent reflections
Radiation source: micro-focus sealed X-ray tube, PhotonJet (Cu) X-ray Source	2341 reflections with *I* > 2σ(*I*)
Mirror monochromator	*R*_int_ = 0.037
Detector resolution: 10.0000 pixels mm^-1^	θ_max_ = 71.4°, θ_min_ = 4.8°
ω scans	*h* = −9→7
Absorption correction: multi-scan (CrysAlis PRO; Rigaku OD, 2020)	*k* = −16→16
*T*_min_ = 0.491, *T*_max_ = 1.000	*l* = −15→15
11239 measured reflections	

### Refinement

**Table:**

Refinement on *F*^2^	Hydrogen site location: mixed
Least-squares matrix: full	H atoms treated by a mixture of independent and constrained refinement
*R*[*F*^2^ > 2σ(*F*^2^)] = 0.023	*w* = 1/[σ^2^(*F*_o_^2^) + (0.0362*P*)^2^ + 0.3283*P*] where *P* = (*F*_o_^2^ + 2*F*_c_^2^)/3
*wR*(*F*^2^) = 0.065	(Δ/σ)_max_ = 0.001
*S* = 1.06	Δρ_max_ = 0.32 e Å^−^^3^
2474 reflections	Δρ_min_ = −0.28 e Å^−^^3^
162 parameters	Extinction correction: SHELXL (Sheldrick, 2015b), Fc^*^=kFc[1+0.001xFc^2^λ^3^/sin(2θ)]^-1/4^
4 restraints	Extinction coefficient: 0.00359 (19)
Primary atom site location: dual	

### Special details

**Table:**

Geometry. All esds (except the esd in the dihedral angle between two l.s. planes) are estimated using the full covariance matrix. The cell esds are taken into account individually in the estimation of esds in distances, angles and torsion angles; correlations between esds in cell parameters are only used when they are defined by crystal symmetry. An approximate (isotropic) treatment of cell esds is used for estimating esds involving l.s. planes.

### Fractional atomic coordinates and isotropic or equivalent isotropic displacement parameters (Å^2^)

**Table:**

	*x*	*y*	*z*	*U*_iso_*/*U*_eq_	
Zn1	0.92145 (3)	0.41694 (2)	0.17409 (2)	0.02862 (11)	
Cl4	0.66523 (6)	0.36967 (4)	0.07893 (3)	0.03361 (13)	
Cl1	0.84550 (6)	0.56627 (3)	0.24448 (4)	0.03547 (13)	
Cl2	0.95542 (7)	0.31035 (3)	0.31521 (4)	0.03674 (14)	
Cl3	1.16765 (6)	0.41294 (4)	0.08236 (4)	0.03691 (14)	
N2	0.4715 (2)	0.39322 (11)	0.28755 (12)	0.0269 (3)	
N1	0.4398 (2)	0.58098 (12)	0.17865 (12)	0.0296 (4)	
C1	0.3676 (2)	0.56492 (13)	0.28262 (13)	0.0245 (4)	
C8	0.3848 (2)	0.47086 (13)	0.33164 (13)	0.0228 (3)	
C9	0.3120 (2)	0.45601 (14)	0.43180 (14)	0.0275 (4)	
C4	0.2233 (2)	0.53608 (16)	0.47875 (15)	0.0340 (4)	
H4	0.173175	0.526950	0.543724	0.041*	
C2	0.2837 (2)	0.64101 (14)	0.33141 (16)	0.0320 (4)	
H2A	0.274830	0.703201	0.299033	0.038*	
C7	0.4940 (3)	0.30526 (14)	0.33447 (16)	0.0335 (4)	
H7	0.556834	0.255296	0.301634	0.040*	
C5	0.3326 (3)	0.36147 (16)	0.47933 (15)	0.0357 (4)	
H5	0.282864	0.349307	0.543880	0.043*	
C6	0.4245 (3)	0.28736 (16)	0.43198 (17)	0.0391 (5)	
H6	0.440294	0.225711	0.464797	0.047*	
C3	0.2103 (3)	0.62624 (16)	0.43029 (16)	0.0367 (5)	
H3	0.152643	0.678533	0.462586	0.044*	
H1A	0.5529 (15)	0.5639 (17)	0.179 (2)	0.042 (7)*	
H1B	0.434 (3)	0.6446 (9)	0.160 (2)	0.056 (8)*	
H1C	0.381 (3)	0.5492 (16)	0.1272 (14)	0.039 (6)*	
H2	0.518 (3)	0.3968 (17)	0.2270 (11)	0.040 (6)*	

### Atomic displacement parameters (Å^2^)

**Table:**

	*U*^11^	*U*^22^	*U*^33^	*U*^12^	*U*^13^	*U*^23^
Zn1	0.02863 (16)	0.02727 (16)	0.03004 (16)	0.00008 (9)	0.00209 (11)	−0.00273 (9)
Cl4	0.0300 (2)	0.0444 (3)	0.0265 (2)	−0.00244 (18)	0.00214 (17)	−0.00831 (18)
Cl1	0.0422 (3)	0.0237 (2)	0.0402 (3)	0.00109 (18)	−0.0005 (2)	−0.00495 (17)
Cl2	0.0478 (3)	0.0272 (2)	0.0354 (2)	0.00672 (19)	0.0037 (2)	0.00296 (17)
Cl3	0.0301 (2)	0.0450 (3)	0.0360 (3)	−0.00615 (18)	0.00597 (19)	−0.00603 (19)
N2	0.0301 (8)	0.0249 (7)	0.0258 (7)	−0.0017 (6)	0.0042 (6)	−0.0003 (6)
N1	0.0371 (9)	0.0273 (9)	0.0244 (8)	−0.0017 (7)	0.0025 (7)	0.0033 (6)
C1	0.0261 (8)	0.0257 (8)	0.0216 (8)	−0.0030 (7)	0.0006 (7)	0.0003 (6)
C8	0.0233 (8)	0.0249 (9)	0.0203 (8)	−0.0030 (7)	−0.0001 (6)	−0.0014 (6)
C9	0.0261 (8)	0.0333 (10)	0.0231 (8)	−0.0076 (7)	0.0009 (7)	0.0000 (7)
C4	0.0305 (9)	0.0469 (12)	0.0250 (9)	−0.0049 (8)	0.0058 (7)	−0.0074 (8)
C2	0.0340 (10)	0.0265 (9)	0.0352 (10)	0.0004 (7)	−0.0006 (8)	−0.0025 (7)
C7	0.0315 (10)	0.0242 (9)	0.0445 (11)	−0.0002 (7)	−0.0007 (8)	0.0014 (8)
C5	0.0367 (10)	0.0440 (11)	0.0266 (9)	−0.0092 (9)	0.0020 (8)	0.0100 (8)
C6	0.0394 (11)	0.0345 (10)	0.0428 (11)	−0.0054 (9)	−0.0041 (9)	0.0161 (9)
C3	0.0337 (10)	0.0391 (11)	0.0376 (11)	0.0012 (8)	0.0053 (8)	−0.0129 (9)

### Geometric parameters (Å, º)

**Table:**

Zn1—Cl4	2.3108 (5)	C8—C9	1.420 (2)
Zn1—Cl1	2.2759 (5)	C9—C4	1.411 (3)
Zn1—Cl2	2.2919 (5)	C9—C5	1.408 (3)
Zn1—Cl3	2.2360 (5)	C4—H4	0.9300
N2—C8	1.362 (2)	C4—C3	1.357 (3)
N2—C7	1.327 (2)	C2—H2A	0.9300
N2—H2	0.860 (10)	C2—C3	1.407 (3)
N1—C1	1.464 (2)	C7—H7	0.9300
N1—H1A	0.882 (10)	C7—C6	1.385 (3)
N1—H1B	0.884 (10)	C5—H5	0.9300
N1—H1C	0.877 (10)	C5—C6	1.366 (3)
C1—C8	1.409 (2)	C6—H6	0.9300
C1—C2	1.364 (3)	C3—H3	0.9300
			
Cl1—Zn1—Cl4	103.058 (19)	C4—C9—C8	118.77 (17)
Cl1—Zn1—Cl2	105.30 (2)	C5—C9—C8	117.93 (18)
Cl2—Zn1—Cl4	107.04 (2)	C5—C9—C4	123.31 (17)
Cl3—Zn1—Cl4	114.479 (19)	C9—C4—H4	119.6
Cl3—Zn1—Cl1	117.08 (2)	C3—C4—C9	120.82 (18)
Cl3—Zn1—Cl2	109.07 (2)	C3—C4—H4	119.6
C8—N2—H2	123.2 (16)	C1—C2—H2A	119.7
C7—N2—C8	123.26 (16)	C1—C2—C3	120.58 (18)
C7—N2—H2	113.6 (16)	C3—C2—H2A	119.7
C1—N1—H1A	111.1 (17)	N2—C7—H7	119.8
C1—N1—H1B	111.1 (18)	N2—C7—C6	120.47 (18)
C1—N1—H1C	113.6 (16)	C6—C7—H7	119.8
H1A—N1—H1B	107 (2)	C9—C5—H5	119.5
H1A—N1—H1C	109 (2)	C6—C5—C9	120.96 (18)
H1B—N1—H1C	105 (2)	C6—C5—H5	119.5
C8—C1—N1	119.84 (15)	C7—C6—H6	120.4
C2—C1—N1	119.86 (16)	C5—C6—C7	119.13 (18)
C2—C1—C8	120.31 (17)	C5—C6—H6	120.4
N2—C8—C1	122.61 (15)	C4—C3—C2	120.33 (18)
N2—C8—C9	118.20 (16)	C4—C3—H3	119.8
C1—C8—C9	119.18 (16)	C2—C3—H3	119.8
			
N2—C8—C9—C4	179.45 (15)	C8—C9—C4—C3	−1.2 (3)
N2—C8—C9—C5	−0.5 (2)	C8—C9—C5—C6	2.0 (3)
N2—C7—C6—C5	0.0 (3)	C9—C4—C3—C2	0.7 (3)
N1—C1—C8—N2	1.9 (2)	C9—C5—C6—C7	−1.8 (3)
N1—C1—C8—C9	−179.12 (16)	C4—C9—C5—C6	−177.92 (18)
N1—C1—C2—C3	178.65 (17)	C2—C1—C8—N2	−178.25 (16)
C1—C8—C9—C4	0.5 (2)	C2—C1—C8—C9	0.7 (3)
C1—C8—C9—C5	−179.45 (16)	C7—N2—C8—C1	177.61 (17)
C1—C2—C3—C4	0.5 (3)	C7—N2—C8—C9	−1.3 (3)
C8—N2—C7—C6	1.6 (3)	C5—C9—C4—C3	178.74 (18)
C8—C1—C2—C3	−1.2 (3)		

### Hydrogen-bond geometry (Å, º)

**Table:**

*D*—H···*A*	*D*—H	H···*A*	*D*···*A*	*D*—H···*A*
N1—H1*A*···Cl1	0.88 (1)	2.31 (1)	3.1309 (16)	154 (2)
N1—H1*B*···Cl2^i^	0.89 (1)	2.39 (1)	3.1747 (17)	148 (2)
N1—H1*C*···Cl3^ii^	0.88 (2)	2.48 (2)	3.2415 (16)	145 (2)
N2—H2···Cl4	0.86 (2)	2.25 (2)	3.0958 (16)	166 (2)
C7—H7···Cl1^iii^	0.93	2.71	3.584 (2)	157

Symmetry codes: (i) −*x*+3/2, *y*+1/2, −*z*+1/2; (ii) *x*−1, *y*, *z*; (iii) −*x*+3/2, *y*−1/2, −*z*+1/2.
